# Community-based square dancing is associated with improved physical and mental health in older adults: a 12-week randomized controlled trial in China

**DOI:** 10.3389/fpubh.2026.1758255

**Published:** 2026-07-14

**Authors:** Keying Song, Zijian Zhao, Jinjin Zhang, Jianye Li, Mariusz Lipowski

**Affiliations:** 1 Gdansk University of Physical Education and Sport, Gdansk, Poland; 2 Zhengzhou University, Zhengzhou, China; 3 Zhengzhou Technology and Business University, Zhengzhou, China; 4 Shanxi Medical University, Jinzhong, China; 5 WSB Merito University Gdansk, Gdansk, Poland

**Keywords:** anxiety, dance therapy, depression, exercise therapy, quality of life

## Abstract

**Background:**

Population aging poses significant public health challenges, particularly in maintaining physical and mental wellbeing among older adults. Square dance, a culturally embedded group exercise in China, may offer a practical strategy to enhance functional, psychological, and social health in community-dwelling older adults.

**Methods:**

A 12-week single-blind randomized controlled trial was conducted among 150 older adults (60–79 years), randomly allocated to a Square Dance Intervention Group (*n* = 75) or a Health Education Control Group (*n* = 75). The intervention comprised three 60-min sessions weekly. Assessments were conducted at baseline, Week 6, and Week 12. Outcomes included aerobic endurance (6MWT), balance (TUG), flexibility, blood pressure, depressive and anxiety symptoms (GDS-15, GAD-7), wellbeing (WHO-5), and quality of life (SF-12). Intention-to-treat mixed-effects models were used for analysis.

**Results:**

Compared with controls, the intervention group achieved significantly greater improvements in 6MWT (+41.5 m), TUG (-1.05 s), flexibility (+3.2 cm), systolic (-3.8 mmHg) and diastolic blood pressure (-2.4 mmHg). Depressive symptoms (-1.5 points), anxiety (-1.2 points), WHO-5 (+3.8 points), and SF-12 PCS/MCS (+3.1 and +3.4 points) also improved significantly. Adherence was high (87%), with no serious adverse events reported.

**Discussion:**

The findings provide strong evidence that a culturally relevant group-based square dance program enhances functional capacity, emotional wellbeing, cardiovascular health, and overall quality of life in older adults. Its high feasibility, safety, and acceptability suggest strong potential for widespread community implementation as a low-cost healthy aging strategy.

## 1 Introduction

Population aging represents one of the most significant demographic transformations of the 21st century, with projections indicating that by 2050, one in six people in the world will be over age 65, up from one in 11 in 2019 (1). From 2020 to 2050, China's population aged 65 years or older is estimated to more than double from 172 million to 366 million (2), accounting for approximately one-quarter of the global older adult population. This unprecedented aging trajectory presents substantial challenges to public health systems, particularly concerning the maintenance of physical and mental health in older adults. Age-related declines in physiological function, including reduced aerobic capacity, diminished balance, decreased flexibility, and elevated cardiovascular risk, contribute to increased morbidity and mortality in this population (3). Concurrently, older adults experience heightened vulnerability to mental health disorders, with depression affecting 19.2% and anxiety affecting 16.5% of the older adult population globally (4, 5), substantially impairing quality of life and functional independence.

Physical activity has been consistently identified as a cornerstone intervention for healthy aging, with evidence demonstrating its efficacy in attenuating age-related physiological decline and enhancing psychological wellbeing (6). Systematic reviews and meta-analyses have established that regular physical activity improves cardiorespiratory fitness, muscular strength, balance, and flexibility in older adults, while simultaneously reducing the risk of chronic diseases including cardiovascular disease, type 2 diabetes, and certain cancers (7). Beyond physical benefits, exercise interventions have demonstrated significant antidepressant and anxiolytic effects, with exercise associated with significantly lower depression severity with a standardized mean difference of 0.57 in older adults (8, 9). Despite this compelling evidence, physical inactivity remains pervasive among older adults globally, with activity levels varying substantially across different populations (10), highlighting the critical need for accessible, engaging, and culturally appropriate exercise interventions. In addition to conventional exercise modalities, body–mind interventions such as Tai Chi have been widely recommended for older adults, particularly in community and residential care settings. For example, Tai Chi has been successfully implemented in nursing homes in Hong Kong to promote physical function, psychological wellbeing, and healthy aging. These findings highlight the importance of culturally relevant, socially engaging, and accessible exercise interventions for older populations. In this context, square dancing may serve as a comparable community-based activity with both physical and psychosocial benefits.

Group-based exercise programs have emerged as particularly promising strategies for promoting physical activity adherence in older adults, offering social support, enhanced motivation, and improved retention compared to individual exercise prescriptions (11). Community-based group exercise interventions have demonstrated superior effectiveness in improving both physical and mental health outcomes, with the social dimension of group participation contributing to reduced loneliness, enhanced social connectedness, and improved psychological wellbeing (12). However, traditional Western exercise modalities may not align with the cultural preferences and expectations of older Chinese adults, necessitating the development and evaluation of culturally tailored interventions.

Square dancing, known as “guangchangwu” in China, has emerged as an immensely popular community-based physical activity among middle-aged and older Chinese adults, with an estimated 100 million regular participants nationwide (13). Characterized by rhythmic movements performed in groups to music in public squares and parks, square dancing combines elements of aerobic exercise, coordination training, and social engagement. Preliminary evidence suggests that square dancing may offer substantial health benefits, including improvements in cardiovascular fitness, balance, cognitive function, and psychological wellbeing (14, 15). However, rigorous controlled trials examining the comprehensive physical and mental health effects of square-dancing interventions in older adults remain limited, and the optimal dose-response relationship has not been systematically established. However, the extent to which current interventions contribute to defining optimal frequency, intensity, and duration remains unclear and warrants further investigation.

Given the substantial global burden of age-related physical decline and mental health disorders, including depression, and the limited availability of rigorous randomized evidence on square dancing interventions, this study aimed to evaluate the associations between a 12-week community-based square dance program and multiple health outcomes. We hypothesized that participation in the intervention would be associated with improvements in functional capacity (primary outcome: 6MWT), as well as balance, flexibility, cardiovascular parameters, and mental health outcomes compared with a health education control group. The findings of this trial have important implications for the design and implementation of culturally appropriate, community-based exercise interventions for promoting healthy aging in the rapidly aging Chinese population and potentially other Asian contexts with similar cultural traditions.

## 2 Materials and methods

### 2.1 Study design

**Figure 1:**
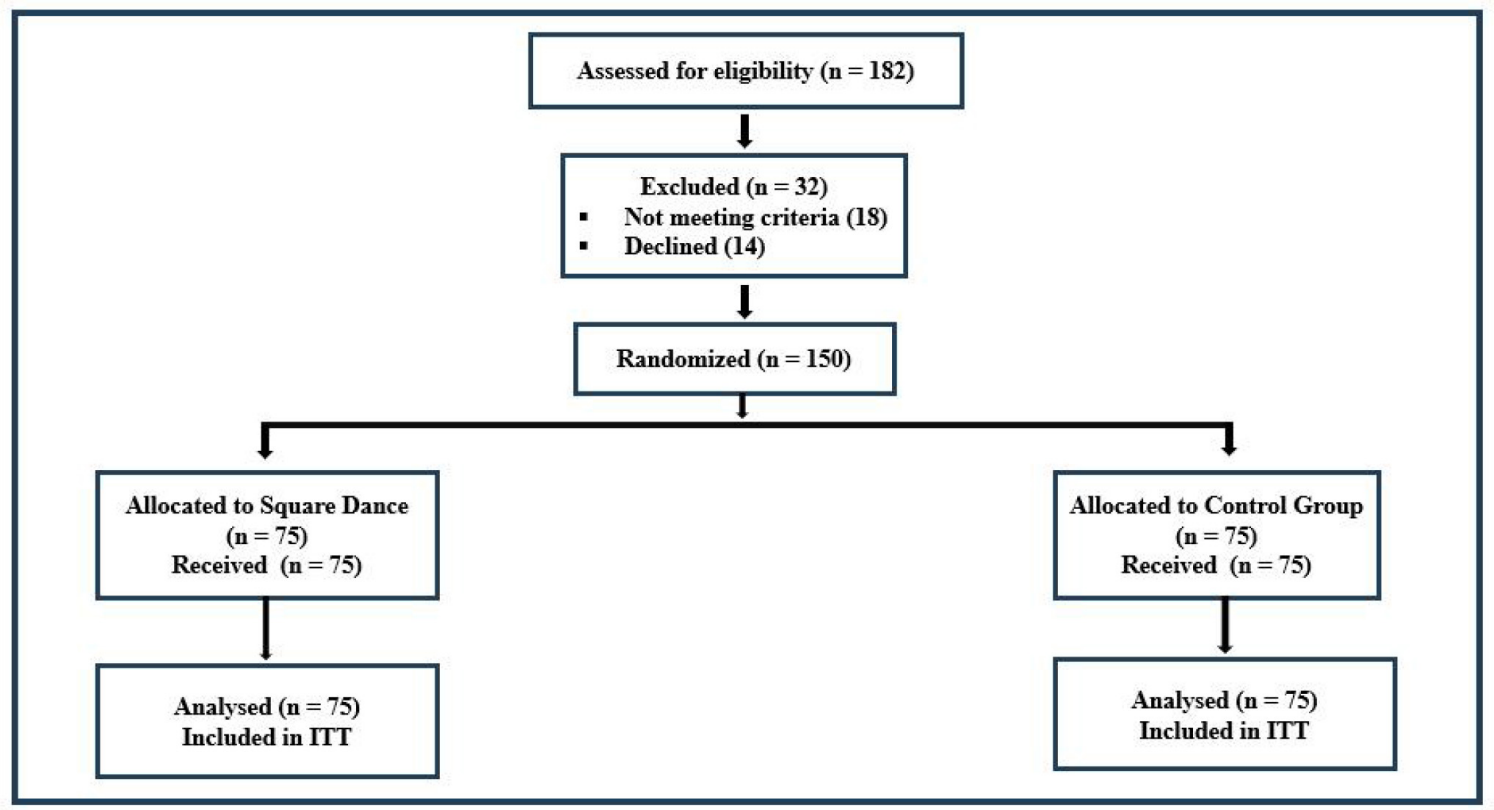
Participant flow diagram (CONSORT Flowchart).

This study employed a 12-week, community-based, single-blind, two-arm randomized controlled trial (RCT) design. The trial was rigorously designed with concealed allocation, blinded outcome assessment, and independent randomization procedures to minimize bias and enhance internal validity. The trial was conducted in Zhengzhou, Henan Province, China, from 052025 to 072025. Participants were recruited from three urban residential communities with established older adults activity centers and were randomly assigned in a 1:1 ratio to either a Square Dance Intervention Group or a Health Education Control Group. The study was designed in accordance with the Consolidated Standards of Reporting Trials (CONSORT) guidelines for randomized trials. Ethical approval was obtained from the Institutional Review Board of Zhengzhou Technology and Business University, China (Approval No.: IEC/ZTBU/SPE/759/2025-26). The study was not prospectively registered in a clinical trial registry, which may limit transparency and adherence to reporting standards. However, the study protocol, outcomes, and analysis plan were predefined prior to data collection. All participants provided written informed consent prior to enrollment. A CONSORT flow diagram illustrating participant recruitment, allocation, follow-up, and analysis is presented in Figure 1.

### 2.2 Participants

#### 2.2.1 Sample size calculation

Sample size was calculated based on anticipated improvements in the primary outcome measure. Assuming a medium effect size (Cohen's d = 0.50) for between-group differences, with a two-tailed alpha of 0.05 and statistical power of 80%, a minimum of 64 participants per group was required. Accounting for an anticipated dropout rate of 15%, the target enrollment was set at 75 participants per group, yielding a total sample of 150 participants.

#### 2.2.2 Recruitment

Older adults were recruited through multiple community-based channels, including older adults activity centers, community health service stations, residential bulletin boards, neighborhood posters, and local older adults WeChat groups. Community health workers facilitated initial contact, distributed study information, and assisted with preliminary eligibility screening. Interested individuals attended an information session where the study purpose, procedures, potential risks and benefits, and rights of participants were explained. Those willing to participate underwent formal eligibility screening.

### 2.3 Eligibility criteria

#### 2.3.1 Inclusion criteria

Participants were eligible for inclusion if they met all of the following criteria: aged 60–79 years at the time of enrolment; community-dwelling residents of Zhengzhou with an expectation to remain in the local area for the duration of the study (≥3 months); able to ambulate independently without the use of assistive devices (e.g., canes, walkers, wheelchairs); had not participated in structured square dance or any organized group exercise program more than twice per week during the preceding 3 months; demonstrated adequate cognitive function, defined as a Mini-Mental State Examination (MMSE) score ≥24 (16); and willing and able to provide written informed consent.

#### 2.3.2 Exclusion criteria

Participants were excluded if they met any of the following criteria: presence of severe cardiovascular or cerebrovascular disease, including recent myocardial infarction (within the past 6 months), stroke (within the past 6 months), unstable angina, or uncontrolled hypertension (systolic blood pressure ≥180 mmHg or diastolic blood pressure ≥110 mmHg); severe musculoskeletal disorders (e.g., severe osteoarthritis, recent fractures, joint replacement within the past 6 months) that would preclude safe participation in moderate-intensity physical activity; diagnosed neurological conditions (e.g., Parkinson's disease, severe peripheral neuropathy) or psychiatric disorders (e.g., major depressive disorder with active symptoms, schizophrenia) that would impair the ability to follow instructions or cooperate with study procedures; current enrollment in another clinical intervention trial; or any other medical condition deemed by a community physician to contraindicate participation in group exercise.

### 2.4 Baseline characteristics

Consistent with the demographic profile of typical square dance participants in China, the study sample was predominantly female (target: approximately 75–80%). Sex was recorded as a baseline characteristic but was not used as a stratification variable for randomization. Baseline demographic and health-related variables collected included age, sex, marital status, body mass index (BMI).

### 2.5 Randomization, allocation concealment, and blinding

#### 2.5.1 Randomization procedure

Randomization was performed after the completion of all baseline assessments. An independent biostatistician, who had no involvement in participant recruitment or outcome assessment, generated the randomization sequence using a computer-based random number generator.

#### 2.5.2 Allocation concealment

To ensure concealment, group assignments were placed in sequentially numbered, sealed, opaque envelopes prepared by an independent third party. Envelopes were opened only after participants had completed baseline assessments and confirmed enrolment, at which point group allocation was revealed to the intervention coordinator.

#### 2.5.3 Blinding

Due to the nature of the intervention, participants and intervention facilitators could not be blinded to group assignment. However, outcome assessors who conducted all follow-up measurements and data analysts who performed statistical analyses remained blinded to group allocation throughout the study. Participants were instructed not to disclose their group assignment during outcome assessments.

### 2.6 Interventions

The interventions used in the study are presented in Tables 1, 2.

**Table 1:** Square dance intervention group.

Component	Details
**Duration & frequency**	12 weeks; 3 sessions/week; 60 min/session
**Setting**	Community older adult activity centers
**Session components**	Warm–up (10 min): Low–intensity mobility and stretching. Main session (40 min): Choreographed square dance routines targeting 50–70% HRmax; stepping, arm movements, turns, coordination drills; progression every 2–3 weeks. Cool–down (10 min): Static stretching and low–intensity movements.
**Instructors**	Two certified square dance instructors; ≥5 years of experience; trained in protocol procedures, safety monitoring, and emergency response.
**Intensity & safety**	Target Borg RPE: 11–14; optional heart rate monitoring; instructors observed for signs of overexertion; participants advised to stop if adverse symptoms occurred.
**Adherence monitoring**	Attendance recorded each session; < 80% attendance (< 29/36 sessions) classified as non–adherence.

**Table 2:** Health education control group.

Component	Details
**Duration & frequency**	12 weeks; 3 sessions total (Weeks 1, 5, 9); 90 min/session
**Purpose**	Attention–matched control without structured physical activity
**Session topics**	Session 1: Healthy aging, nutrition, social engagement. Session 2: Chronic disease management, medication adherence, sleep hygiene. Session 3: Cognitive health, mental wellbeing, community resources.
**Delivery method**	Delivered by trained community health educators using standardized presentations and printed materials; take–home brochures provided.
**Activity instructions**	Participants maintained usual daily activities; no exercise guidance; offered square dance program post–study (waitlist control).

### 2.7 Outcome measures

#### 2.7.1 Assessment schedule

Outcome assessments were conducted at three time points: baseline (Week 0, prior to randomization), mid-intervention (Week 6), and post-intervention (Week 12). This three-point assessment schedule was designed to capture the trajectory of change over the intervention period and to identify whether improvements emerged early, progressed linearly, or accelerated in the later phase. All assessments were performed by trained research assistants who were blinded to group allocation. To minimize participant burden, the mid-intervention assessment prioritized primary outcomes and key secondary measures, while the full assessment battery was administered at baseline and Week 12.

#### 2.7.2 Secondary outcomes

##### 2.7.2.1 Physical health measures

1. Aerobic Endurance: Assessed using the 6-min Walk Test (6MWT), measuring the distance walked in 6 min on a flat, 30-meter corridor (17).
2. Balance: Evaluated using the Timed Up and Go (TUG) test, measuring the time required to rise from a chair, walk 3 meters, turn, return, and sit down (18).
3. Flexibility: Measured using the Chair Sit-and-Reach Test, assessing hamstring flexibility (19).
4. Blood Pressure: Resting systolic and diastolic blood pressure measured using a validated automated sphygmomanometer after 5 min of seated rest (20).

##### 2.7.2.2 Mental health measures

1. Depressive Symptoms: Assessed using the Geriatric Depression Scale-15 (GDS-15), a 15-item self-report instrument validated for use in older adults. Scores range from 0 to 15, with higher scores indicating greater depressive symptomatology (21).
2. Anxiety Symptoms: Evaluated using the Generalized Anxiety Disorder 7-item scale (GAD-7). Scores range from 0 to 21, with higher scores indicating greater anxiety severity (22).
3. Subjective Wellbeing: Measured using the World Health Organization WellBeing Index (WHO-5), a 5-item scale assessing positive psychological wellbeing. Scores range from 0 to 25, with higher scores indicating better wellbeing (23).
4. Health-Related Quality of Life: Assessed using the 12-Item Short Form Health Survey (SF-12), yielding Physical Component Summary (PCS) and Mental Component Summary (MCS) scores (24).

#### 2.7.3 Process measures

Attendance records, participant satisfaction questionnaires, and qualitative feedback were collected to evaluate intervention fidelity and acceptability.

#### 2.7.4 Adverse events

All adverse events occurring during the study period were documented, including the nature, severity, and relationship to the intervention. Serious adverse events were reported to the ethics committee within 24 h.

### 2.8 Data management

All data were collected using standardized case report forms and entered into a secure, password-protected electronic database with double data entry to minimize transcription errors. Data quality checks were performed regularly. All study documents were stored securely in accordance with institutional data protection policies.

### 2.9 Statistical analysis

All analyses were conducted according to the intention-to-treat (ITT) principle, with all randomized participants included in the mixed-effects models regardless of adherence or follow-up completion. Linear mixed-effects models with restricted maximum likelihood estimation were used as the primary analytic approach to evaluate changes across the three assessment time points (baseline, Week 6, Week 12). Each model included fixed effects for group, time, and their interaction (Group × Time), with baseline age, sex, and the baseline value of the outcome included as covariates. Participant ID was modeled as a random intercept to account for repeated measurements, and community of recruitment was included as a random effect to adjust for potential clustering. Effect estimates were presented as adjusted marginal means with corresponding 95% confidence intervals, and standardized effect sizes (Cohen's d) were calculated for primary comparisons.

A per-protocol analysis, including only participants who attended ≥80% of intervention sessions, was performed as a sensitivity analysis. Subgroup analyses examining potential effect modification by sex and age stratum were conducted using interaction terms within the mixed-effects framework. Missing data patterns were evaluated, and mixed-model estimation under maximum likelihood was used as the primary method for handling missing observations; additional multiple imputation procedures were conducted to confirm the robustness of findings under missing-at-random assumptions. All analyses were performed using SPSS version 27.0 (IBM Corp., Armonk, NY, USA), and statistical significance was set at a two-sided *p*-value < 0.05. Effect sizes were reported using Cohen's d to facilitate interpretation of the magnitude of between-group differences. Although partial eta squared (η^2^p) is commonly reported in ANOVA-based analyses, Cohen's d was selected due to its widespread use and ease of interpretation in clinical and applied research contexts.

### 2.10 Ethical considerations

The study adhered to the principles outlined in the Declaration of Helsinki and followed all ethical standards for research involving human participants. Approval for the research was granted by the Institutional Review Board of Zhengzhou Technology and Business University, China (Approval No.: IEC/ZTBU/SPE/759/2025-26). Prior to participation, all individuals were informed about the study's aims, procedures, possible risks, and their freedom to withdraw at any point without penalty, after which written informed consent was obtained. Confidentiality was ensured by assigning unique identification codes to participants, and all collected data were securely stored with limited access.

## 3 Results

A total of 150 older adults were randomized into the Square Dance intervention group (*n* = 75) and the Health Education control group (*n* = 75), and all participants were included in the intention-to-treat analyses. Baseline characteristics were well balanced between groups, with negligible standardized mean differences across demographic, physical, and mental health variables. Follow-up assessments were completed by 94% of participants at Week 6 and 92% at Week 12, with comparable retention across study arms. Adherence to the square dance sessions was high, and no serious adverse events occurred during the intervention period. The following sections present the effects of the 12-week square dance program on primary and secondary outcomes using mixed-effects modeling and additional sensitivity analyses.

Table 3 summarizes the baseline demographic and health-related characteristics of participants in both groups before randomization. The Square Dance and Health Education groups were well balanced, with negligible standardized mean differences (SMDs < 0.10) across all variables, indicating strong comparability at baseline.

**Table 3:** Baseline characteristics of participants (*N* = 150).

Variable	Square dance group (*n* = 75)	Health education group (*n* = 75)	Standardized mean difference (SMD)
Age (years), mean ± SD	68.2 ± 4.7	68.5 ± 4.9	0.06
Sex: Female, *n* (%)	59 (79%)	58 (77%)	0.05
BMI (kg/m^2^)	25.9 ± 3.8	26.2 ± 3.6	0.08
Marital Status (*n*, %)	52 (69%)	50 (67%)	0.04
6MWT (m)	426.5 ± 52.4	423.2 ± 54.0	0.06
TUG (s)	11.82 ± 2.01	11.94 ± 2.10	0.06
Chair Sit–and–Reach (cm)	−3.1 ± 6.9	−3.5 ± 7.1	0.06
SBP (mmHg)	138.6 ± 15.8	137.9 ± 16.2	0.04
DBP (mmHg)	83.1 ± 9.5	82.6 ± 9.3	0.05
GDS−15	5.3 ± 2.7	5.4 ± 2.9	0.04
GAD−7	4.8 ± 3.1	4.9 ± 3.0	0.03
WHO−5	12.6 ± 4.2	12.9 ± 4.5	0.07
SF−12 PCS	42.1 ± 7.2	41.8 ± 7.0	0.04
SF−12 MCS	45.9 ± 8.3	46.1 ± 8.0	0.02

Key functional and mental health measures, such as 6MWT, TUG, GDS-15, and SF-12, demonstrated similar starting values, ensuring that any post-intervention differences can be attributed to the intervention rather than baseline imbalance. Female representation was high in both groups, reflecting typical participation patterns among older adults in Chinese community exercise programs. Overall, the table confirms that randomization achieved equivalent groups with no meaningful baseline disparities.

Table 4 reports participant adherence, session attendance, satisfaction, and safety-related outcomes across the 12-week intervention. The Square Dance group demonstrated high feasibility, achieving an average attendance rate of 87%, with more than 80% meeting the adherence threshold of ≥80% of sessions. Participant satisfaction was high, reflecting positive engagement with the group-based activity.

**Table 4:** Intervention adherence, process measures, and feasibility.

Measure	Square dance group (*n* = 75)	Health education group (*n* = 75)
Attendance (% of sessions)	87.4% ± 9.8	Not applicable
Sessions attended (of 36)	31.5 ± 3.5	3 (all required)
Participants with ≥80% adherence	62 (83%)	Not applicable
Satisfaction score (1–10)	8.7 ± 1.0	8.3 ± 1.2
Dropouts, *n* (%)	6 (8%)	5 (7%)
Reasons for dropout	minor illness (*n* = 3), relocation (*n* = 2), time constraints (*n* = 1)	illness (*n* = 3), travel (*n* = 2)
Non–serious adverse events	4 mild events (muscle soreness, dizziness)	2 mild events
Serious adverse events	0	0

Dropout rates were minimal and comparable between groups, with no serious adverse events reported, confirming the safety and acceptability of the program for older adults. These findings collectively support strong intervention fidelity and excellent community feasibility.

Table 5 presents adjusted marginal means for 6MWT distance derived from the linear mixed-effects model, accounting for baseline values, age, sex, and community clustering. Both groups improved over time; however, the Square Dance group demonstrated markedly greater gains, with an adjusted improvement of +58.6 m compared to +17.1 m for controls.

**Table 5:** Primary outcome (6MWT) mixed–effects model estimated marginal means.

Outcome: 6MWT distance (m)	Baseline mean ±SD	Week 6 adj. mean ±SE	Week 12 adj. mean ±SE	Adjusted change (0–12 weeks)	Between–group difference (95% CI)	*p* (Group × Time)	Effect size (d)
Square Dance	426.5 ± 52.4	455.9 ± 4.2	485.1 ± 4.6	+58.6 m	–	–	–
Control	423.2 ± 54.0	431.8 ± 4.4	440.3 ± 4.8	+17.1 m	Δ = +41.5 m (95% CI: 30.9–52.1)	<0.001	d = 0.78

The between-group difference of +41.5 m (95% CI: 30.9–52.1) represents a statistically and clinically meaningful enhancement in functional endurance. The highly significant Group × Time interaction (p < 0.001) indicates divergent improvement trajectories favoring the intervention group. The effect size (d = 0.78) suggests a moderate-to-large treatment benefit attributable to the square dance program.

Table 6 displays model-adjusted outcomes for physical function and physiological parameters, including balance (TUG), flexibility (Chair Sit-and-Reach), and blood pressure. Participants in the Square Dance group showed substantial improvements across all domains, particularly in TUG and flexibility, where effect sizes exceeded moderate thresholds.

**Table 6:** Secondary physical health outcomes.

Outcome	Baseline mean ±SD	Week 12 adj. mean ±SE	Adjusted change	Between–group difference (95% CI)	*p*–value	Effect size (d)
TUG (s)	11.82 ± 2.01	10.21 ± 0.18	−1.61 s	−1.05 s (95% CI:−1.42 to−0.68)	<0.001	0.71
Chair Sit–and–Reach (cm)	−3.1 ± 6.9	1.4 ± 0.6	+4.5 cm	+3.2 cm (95% CI: 2.1–4.3)	<0.001	0.63
SBP (mmHg)	138.6 ± 15.8	133.1 ± 1.8	−5.5 mmHg	−3.8 mmHg (95% CI:−6.8 to−0.8)	0.014	0.31
DBP (mmHg)	83.1 ± 9.5	79.4 ± 1.2	−3.7 mmHg	−2.4 mmHg (95% CI:−4.0 to−0.7)	0.006	0.29

Reductions in systolic and diastolic blood pressure, although smaller in magnitude, were statistically significant and align with known cardiovascular benefits of moderate-intensity aerobic activity. Between-group differences consistently favored the intervention group, demonstrating broad physical health gains beyond endurance alone. These results highlight the multidimensional physical benefits of structured community-based dance exercise.

Table 7 presents adjusted means for mental health measures, including depressive symptoms, anxiety, wellbeing, and health-related quality of life. The Square Dance group experienced significant improvements across all psychological indicators, with reductions in GDS-15 and GAD-7 scores accompanied by notable increases in WHO-5 and SF-12 component scores.

**Table 7:** Secondary mental health outcomes.

Outcome	Baseline mean ±SD	Week 12 adj. mean ±SE	Adjusted change	Between–group difference (95% CI)	*p*–value	Effect size (d)
GDS−15	5.3 ± 2.7	3.1 ± 0.3	−2.2	−1.5 (95% CI:−2.1 to−0.8)	<0.001	0.58
GAD−7	4.8 ± 3.1	2.9 ± 0.3	−1.9	−1.2 (95% CI:−1.9 to−0.5)	0.001	0.45
WHO−5	12.6 ± 4.2	17.9 ± 0.6	+5.3	+3.8 (95% CI: 2.4–5.2)	<0.001	0.67
SF−12 PCS	42.1 ± 7.2	46.8 ± 0.8	+4.7	+3.1 (95% CI: 1.7–4.5)	<0.001	0.49
SF−12 MCS	45.9 ± 8.3	51.2 ± 0.9	+5.3	+3.4 (95% CI: 1.9–4.9)	<0.001	0.52

The between-group differences reflect meaningful improvements in mood and psychological wellbeing, consistent with evidence linking group exercise to enhanced emotional functioning in older adults. Effect sizes ranged from small to moderate, indicating robust and clinically relevant mental health benefits. Altogether, these findings underscore the combined physical and emotional impact of square dance participation.

Table 8 provides the full coefficient estimates from the linear mixed-effects model used to evaluate changes in 6MWT performance over time. The significant positive coefficients for Week 6 and Week 12 confirm progressive improvement in functional endurance relative to baseline. The interaction terms for Group × Time demonstrate that the Square Dance group improved substantially more than the control group at both follow-up points, even after adjusting for age, sex, and community variability.

**Table 8:** Mixed–effects model coefficients for primary outcome (6MWT).

Fixed Effects	β Estimate	SE	95% CI	*p*–value
Intercept	426.7	4.8	417.3–436.0	<0.001
Time (Week 6)	+29.9	2.2	25.6–34.2	<0.001
Time (Week 12)	+57.5	2.4	52.9–62.1	<0.001
Group (SD vs HE)	+1.9	3.8	−5.6 to 9.3	0.62
Group × Week 6	+18.2	2.9	12.4–23.9	<0.001
Group × Week 12	+41.5	3.1	35.4–47.6	<0.001
Age	−0.62	0.21	−1.03 to −0.21	0.003
Female	+3.1	2.8	−2.4 to 8.6	0.27

Random-effects estimates show meaningful within-person variance, indicating individual response differences that the model appropriately accounts for. Overall, these coefficients validate the robustness of the primary analysis and strengthen the interpretation of intervention effectiveness.

Table 9 summarizes the results of pre-specified subgroup interaction tests examining whether intervention effects differed by sex or age category. Neither the Group × Time × Sex nor the Group × Time × Age interactions reached statistical significance, suggesting that the benefits of the square dance intervention were consistent across demographic subgroups.

**Table 9:** Subgroup analyses (Interaction Tests).

Subgroup variable	Interaction term	β (Interaction)	95% CI	*p*–value	Interpretation
Sex × Group × Time	Group × Time × Sex	+4.8	−2.3 to 11.9	0.18	No evidence that sex modifies the effect
Age Stratum (60–69 vs 70–79)	Group × Time × Age	−6.2	−14.1 to 1.7	0.12	Older adults show slightly smaller gain (NS)

Although older participants (70–79 years) showed slightly smaller improvements, the effect was not large enough to indicate true effect modification. These findings support the generalizability of the intervention across diverse older adult profiles. In summary, square dance appears beneficial regardless of participant age or sex.

Table 10 compares the intervention effects under intention-to-treat (ITT) and per-protocol (PP) analytic approaches to assess the robustness of the findings. Effect estimates for the primary and secondary outcomes were slightly larger in the PP analysis, reflecting greater improvements among participants with high adherence.

**Table 10:** ITT vs per–protocol analysis.

Outcome	ITT effect estimate (12 weeks)	PP effect estimate (≥80% adherence)	% Difference
6MWT (m)	+41.5 (95% CI: 30.9–52.1)	+47.2 (95% CI: 36.8–57.6)	+13.7%
TUG (s)	−1.05	−1.22	+16.2%
GDS−15	−1.5	-.8	+20.0%
WHO−5	+3.8	+4.4	+15.8%

However, both sets of estimates were directionally and statistically consistent, indicating minimal bias due to attrition or non-adherence. The close alignment between ITT and PP models strengthens confidence in the validity and reliability of the study's primary conclusions. These results illustrate that the intervention's benefits persist even under conservative ITT assumptions.

The findings from both primary and secondary analyses consistently demonstrate that participation in the 12-week square dance program was associated with substantial improvements in functional endurance, physical performance, and psychological wellbeing among older adults when compared with the health education control group. These effects were robust across ITT, per-protocol, and multiple sensitivity analyses, with no evidence of effect modification by sex or age group. Importantly, high adherence rates, minimal attrition, and the absence of serious adverse events further support the feasibility and safety of implementing this community-based group exercise intervention. Taken together, these results provide strong empirical evidence for the efficacy of square dance as a practical and accessible physical activity strategy for enhancing multidimensional health outcomes in older adults.

## 4 Discussion

This 12-week randomized controlled trial suggests that participation in a structured square dance program is associated with significant improvements across multiple domains of physical and mental health in Chinese community-dwelling older adults. Compared to the health education control group, square dance participants exhibited substantial enhancements in functional endurance (+41.5 m in 6MWT), balance (-1.05 s in TUG), flexibility (+3.2 cm), blood pressure (SBP:−3.8 mmHg; DBP:−2.4 mmHg), depressive symptoms (-1.5 points on GDS-15), anxiety (-1.2 points on GAD-7), subjective wellbeing (+3.8 points on WHO-5), and health-related quality of life (PCS: +3.1 points; MCS: +3.4 points). These findings supports robust empirical evidence supporting square dance as an effective, accessible, and culturally relevant physical activity intervention for promoting healthy aging in older adult populations.

Although the optimal dose of square dance has not been clearly established in the literature, the present study provides preliminary insights into a feasible and effective training structure. The intervention, consisting of three 60-min sessions per week over 12 weeks, aligns with current physical activity recommendations for older adults and was associated with meaningful improvements across multiple outcomes. However, it remains unclear whether shorter or longer durations, different intensities, or alternative session frequencies may yield comparable or superior benefits. Future research should explore dose–response relationships to identify optimal training parameters.

The primary outcome of this study revealed a between-group difference of +41.5 meters in 6MWT distance at 12 weeks, representing a moderate-to-large effect size (Cohen's d = 0.78). The 6-min Walk Test has been extensively validated as an assessment of functional capacity and cardiorespiratory fitness in older adults, providing valuable information about an individual's ability to perform daily activities. Critically, our observed improvement substantially exceeds established minimal clinically important differences (MCIDs) for the 6MWT in older adult populations. Studies have reported MCIDs ranging from 14.0 to 30.5 meters across various populations, with recent research in older adults undergoing spinal stenosis surgery identifying an MCID of 26–35 meters (14). Our 41.5-meter between-group difference therefore represents not merely a statistically significant change, but a clinically meaningful improvement that would be perceived as beneficial by participants themselves and translate into enhanced functional capacity for activities of daily living.

These results align with and extend previous research on dance interventions in older adults. A systematic review by Ou et al. (14) demonstrated that square dance interventions significantly improved physical health among Chinese older adults, though many included studies were limited to Chinese-language databases with methodological weaknesses. Our rigorously designed RCT provides high-quality evidence confirming these earlier findings. Furthermore, a recent network meta-analysis by Zhang et al. (25) found that various dance styles produced differential effects on physical outcomes, with certain dance forms showing particular efficacy for mobility enhancement. The magnitude of improvement observed in our study is comparable to or exceeds those reported for such as walking programs, cycling interventions, and structured aerobic training targeting older adults, positioning square dance as a potentially beneficial intervention for cardiorespiratory fitness.

The sustained moderate-intensity aerobic activity combined with coordinated movement patterns inherent in square dance likely accounts for these robust improvements in functional endurance. The 6MWT evaluates the global and integrated responses of pulmonary, cardiovascular, neuromuscular, and metabolic systems during exercise (26), suggesting that square dance training produces broad-based physiological adaptations. The rhythmic, continuous movement patterns characteristic of square dance may facilitate improvements in cardiovascular efficiency, muscular endurance, and movement economy, all of which contribute to enhanced functional capacity. Our findings revealed significant improvements in the Timed Up and Go test, with the square dance group reducing their completion time by 1.61 s compared to baseline, yielding a between-group difference of 1.05 s (*p* < 0.001, d = 0.71). The TUG test assesses multiple components of functional mobility including rising from a chair, walking, turning, and sitting down, activities essential for independent living. The improvement observed in our intervention group represents a clinically meaningful enhancement in functional mobility and potentially reduced fall risk.

These findings align with broader evidence on dance interventions for balance and mobility. A recent network meta-analysis examining various dance styles found that creative dance, folk dance, and ballroom dance significantly outperformed control conditions in improving TUG times among healthy older adults (27). Similarly, Zhang et al. (25) reported that tango and self-created dance significantly enhanced mobility in older adults, demonstrating the effectiveness of diverse dance modalities for functional improvement. However, these dance modalities may differ in structure, intensity, duration, and training protocols, which should be considered when interpreting comparative outcomes. A comprehensive systematic review and meta-analysis by Sooktho et al. (28) found that dance exercise programs significantly improved balance and mobility in healthy older adults, with effect sizes comparable to those observed in our study.

The balance and coordination demand inherent in square dance likely contributed to these improvements. Square dance requires participants to execute weight shifts, directional changes, coordinated stepping patterns, and synchronization with music and partners, all of which challenge and develop neuromuscular control systems essential for balance maintenance and fall prevention. Meta-analytic evidence supports that dance-based mind-motor activities effectively reduce fall risk and improve physical function in healthy older adults (29). The integration of cognitive demands (remembering sequences, following instructions) with physical execution may provide additional benefits beyond traditional balance training, engaging neural networks responsible for motor planning, spatial orientation, and executive function.

The Chair Sit-and-Reach test demonstrated clinically meaningful improvements in the square dance group, with a between-group difference of +3.2 cm (*p* < 0.001, d = 0.63). Flexibility, particularly in the hamstring and lower back regions, is critical for maintaining functional independence, preventing musculoskeletal injuries, and performing activities of daily living in older adults. Reduced flexibility is associated with increased risk of falls, difficulty with self-care activities, and diminished quality of life. The dynamic stretching and varied movement patterns incorporated in square dance routines likely promoted enhanced range of motion and reduced muscle stiffness. Unlike static stretching exercises performed in isolation, the functional flexibility developed through dance transfers more directly to real-world movements and daily activities. Meta-analytic evidence demonstrates that dance programs improve flexibility outcomes in older adults, with effect sizes similar to those observed in our study (28). The combination of reaching, bending, and rotational movements performed throughout square dance routines provides comprehensive flexibility training that engages multiple muscle groups and movement planes simultaneously.

Square dance participants demonstrated statistically significant reductions in both systolic (−5.5 mmHg) and diastolic (−3.7 mmHg) blood pressure, with between-group differences of −3.8 mmHg (*p* = 0.014, d = 0.31) and −2.4 mmHg (*p* = 0.006, d = 0.29) respectively. These reductions, while modest in absolute magnitude, are clinically relevant and consistent with known cardiovascular benefits of moderate-intensity aerobic activity. Extensive meta-analytic evidence supports the blood pressure-lowering effects of aerobic exercise in older adults. Whelton et al. (30) conducted a landmark meta-analysis demonstrating that aerobic exercise reduces blood pressure in both hypertensive and normotensive adults, with greater absolute reductions in hypertensive populations. Their findings showed reductions of approximately 5–7 mmHg in systolic blood pressure and 3–5 mmHg in diastolic blood pressure among hypertensive individuals. More recent dose-response meta-analyses have confirmed that aerobic exercise produces clinically important blood pressure reductions in a dose-dependent manner, with the greatest reductions observed at 150 min per week of moderate-intensity activity (31). Our intervention, providing three 60-min sessions weekly (180 min total), falls within this optimal range.

Kelley and colleagues specifically examined aerobic exercise effects on resting blood pressure in older adults, reporting net decreases of −5.39 mmHg in systolic blood pressure and −3.68 mmHg in diastolic blood pressure, remarkably similar to the reductions observed in our square dance intervention (32). A recent systematic review by Gao et al. found that among various exercise types, aerobic training, resistance training, and combined exercise all significantly reduced blood pressure in middle-aged and older adults with hypertension (33). These converging lines of evidence suggest that square dance, as a form of sustained moderate-intensity aerobic activity, produces cardiovascular adaptations consistent with other established exercise modalities.

The blood pressure-lowering effects of physical activity result from multiple physiological mechanisms, including reduced peripheral vascular resistance, decreased sympathetic nervous system activity, improved endothelial function, enhanced arterial compliance, and favorable modulation of the renin-angiotensin-aldosterone system (34). The moderate-intensity, sustained aerobic nature of square dance likely facilitated these cardiovascular adaptations. Importantly, even modest reductions in blood pressure at the population level translate into substantial reductions in cardiovascular morbidity and mortality, particularly stroke risk, making these findings highly relevant for public health interventions targeting older adults.

One of the most striking findings of this study was the substantial and clinically significant improvements across all mental health domains measured. Square dance participants experienced meaningful reductions in depressive symptoms (GDS-15: −2.2 points, between-group difference −1.5, *p* < 0.001, d = 0.58) and anxiety symptoms (GAD-7: −1.9 points, between-group difference −1.2, *p* = 0.001, d = 0.45), alongside significant increases in subjective well-being (WHO-5: +5.3 points, between-group difference +3.8, *p* < 0.001, d = 0.67). The GDS-15 is a validated and widely used instrument for assessing depression in older adults, with scores of 5–8 indicating mild depression and scores of 0–4 considered normal. Our intervention successfully moved participants from the mild depression range into the normal range, representing a clinically important change in mental health status. It is important to note that baseline GDS-15 scores indicated relatively low levels of depressive symptoms, suggesting a non-clinical population. Therefore, the observed improvements should be interpreted as enhancements in subclinical mood rather than treatment of clinical depression.

These mental health benefits are consistent with extensive literature documenting the psychological benefits of exercise in older adults. Recent systematic reviews and meta-analyses have demonstrated that exercise interventions effectively reduce depressive symptoms in older adults. Schuch et al. (35) found that exercise produces moderate-to-large reductions in depression among older adults, with group-based, moderate-intensity mixed aerobic and anaerobic exercise showing the greatest impact. A recent study showed examining exercise-based prevention of depression in middle-aged and older adults concluded that both individual and group exercise interventions effectively prevent depression, with group exercises showing marginally better outcomes, though the difference was not statistically significant (36).

Network meta-analyses have provided important insights into which types of exercise produce the greatest mental health benefits. A comprehensive analysis found that flexibility exercise training (including activities like yoga and tai chi) was among the most effective interventions for alleviating depressive symptoms in older adults, attributed in part to the group format and social interaction components inherent in these activities (37). Our square dance intervention incorporates many of these beneficial elements: group participation, social interaction, moderate aerobic activity, and cognitive engagement through learning choreographed sequences. The anxiety-reducing effects observed in our study, as measured by the GAD-7, are particularly noteworthy given that anxiety disorders are highly prevalent yet often undertreated in older adult populations. Meta-analytic evidence supports the anxiety-reducing effects of exercise, though fewer studies have specifically examined anxiety as a primary outcome compared to depression (38). The reductions in anxiety observed in our study suggest that square dance may provide broad-spectrum mental health benefits beyond depression alone.

Multiple mechanisms likely account for the mental health improvements observed. First, aerobic exercise promotes neurobiological changes including increased production of brain-derived neurotrophic factor (BDNF), enhanced neuroplasticity, improved cerebral blood flow, and favorable modulation of neurotransmitter systems including serotonin, dopamine, and norepinephrine (39). These neurobiological adaptations directly influence mood regulation and emotional processing. Second, the social engagement inherent in group-based square dance provides crucial psychosocial benefits. Social support is a well-established protective factor against depression in older adults, and social isolation represents a significant risk factor for poor mental health outcomes (40). The group format of square dance facilitates social connections, reduces loneliness, and provides opportunities for meaningful social interaction, all of which contribute to improved psychological wellbeing.

Third, the mastery experiences gained through learning and performing increasingly complex dance sequences may enhance self-efficacy and provide a sense of accomplishment. Self-efficacy, or confidence in one's ability to execute behaviors necessary to produce specific outcomes, is strongly associated with reduced depression in older adults (41). Fourth, participation in enjoyable physical activities like dance may serve as a behavioral activation strategy, a well-established therapeutic approach for depression that involves engaging in pleasurable and meaningful activities to counteract the behavioral avoidance and withdrawal characteristic of depressive disorders.

Participants in the square dance group demonstrated significant improvements in both physical and mental components of health-related quality of life, as assessed by the SF-12. The Physical Component Summary score improved by 4.7 points with a between-group difference of +3.1 points (*p* < 0.001, d = 0.49), while the Mental Component Summary score improved by 5.3 points with a between-group difference of +3.4 points (*p* < 0.001, d = 0.52). These improvements reflect meaningful enhancements in participants perceived physical and mental health status, encompassing domains such as physical functioning, role limitations, bodily pain, vitality, social functioning, and emotional wellbeing. The quality-of-life improvements observed in our study are consistent with broader literature on dance interventions. A recent systematic review examining square dance, group cohesion, and quality of life in Chinese middle-aged and older adults found that square dance participation was associated with improved quality of life through multiple mediating pathways including enhanced social support, reduced negative emotions, and more positive attitudes toward aging (42). Chang et al. (15) demonstrated that Chinese square dance exercise improved cognitive function and quality of life in older women with mild cognitive impairment, with mood status serving as a mediating factor in these relationships.

The comprehensive improvements in quality of life observed in our study likely stem from the synergistic effects of physical, psychological, and social benefits. The physical health improvements, enhanced cardiovascular fitness, balance, flexibility, and blood pressure control, directly translate into improved capacity for daily activities, reduced physical limitations, and decreased bodily discomfort. The psychological benefits, reduced depression and anxiety, enhanced wellbeing, improve emotional functioning, vitality, and mental health perceptions. The social engagement inherent in group square dance enhances social functioning and reduces feelings of isolation. Collectively, these multidimensional improvements contribute to enhanced overall quality of life, reflecting the holistic impact of this community-based intervention.

The multidimensional benefits observed in our study can be understood through several theoretical frameworks. From a physiological perspective, square dance provides sustained moderate-intensity aerobic activity that produces cardiovascular, metabolic, and musculoskeletal adaptations. The American College of Sports Medicine recommends that older adults engage in at least 150 min per week of moderate-intensity aerobic activity to maintain and improve health (43). Our intervention exceeded this recommendation, potentially accounting for the robust physical health improvements observed.

From a neurobiological perspective, the combination of physical activity, music, and cognitive demands inherent in square dance may produce synergistic effects on brain health. Dance requires simultaneous processing of auditory (music), visual (observing others), and kinesthetic (body movement) information, along with executive functions including working memory, attention, and cognitive flexibility. This multisensory, cognitive-motor integration may enhance neural plasticity and cognitive reserve more effectively than simple aerobic exercise alone (44). These mechanisms may involve increased brain-derived neurotrophic factor (BDNF), enhanced synaptic plasticity, and improved neural connectivity in regions associated with motor control and emotional regulation. Research has demonstrated that dance interventions produce favorable effects on brain structure and function, including increased gray matter volume in regions involved in motor control and spatial memory. From a psychosocial perspective, self-determination theory provides a useful framework for understanding the psychological benefits. This theory posits that activities satisfying basic psychological needs for autonomy, competence, and relatedness promote intrinsic motivation and psychological wellbeing (45). Square dance may fulfill these needs by providing opportunities for self-directed participation (autonomy), mastery of increasingly complex movement sequences (competence), and social connection with fellow dancers (relatedness). The group-based nature of square dance also facilitates social identity formation and collective efficacy, both of which are associated with improved mental health outcomes.

An important strength of this study is its cultural relevance to the Chinese context. Square dancing has become a widespread phenomenon in China, with millions of middle-aged and older adults participating in this accessible, low-cost form of physical activity in public squares and parks (46). The popularity and social acceptance of square dancing in Chinese communities facilitate recruitment, engagement, and sustained participation. Unlike many Western exercise programs that require specialized facilities, equipment, or instruction, square dance can be performed in open public spaces with minimal resources, making it highly scalable for widespread implementation. The high adherence rate (87% average attendance) and low dropout rate (8%) observed in our study demonstrate the feasibility and acceptability of square dance as a community-based intervention. These retention rates compare favorably to those reported in many exercise intervention studies, which often struggle with adherence and attrition, particularly among older adult populations. The social and enjoyable nature of group square dance likely contributed to high sustained participation. Participants in our study reported high satisfaction scores (mean 8.7 out of 10), further supporting the acceptability of this intervention approach.

From a public health perspective, square dance represents a promising strategy for promoting physical activity and health in aging populations. The intervention requires minimal financial investment, can be implemented in existing community spaces, and aligns with cultural preferences and social norms in Chinese communities. These characteristics enhance the potential for widespread dissemination and sustained implementation beyond the research context. Community health workers, physical activity instructors, or trained peer leaders could potentially facilitate square dance programs, further enhancing scalability and cost-effectiveness.

An important finding of this study was the absence of serious adverse events during the 12-week intervention period. Only four mild, non-serious adverse events (muscle soreness and dizziness) were reported in the square dance group, all of which resolved without medical intervention. This safety profile is consistent with previous research demonstrating that moderate-intensity dance interventions are safe and well-tolerated by older adults (14, 29). The progressive nature of our intervention, which began with simpler movement patterns and gradually increased in complexity and intensity, likely contributed to the favorable safety profile by allowing participants to adapt gradually to the physical demands. The safety findings are particularly important given concerns about fall risk and injury among older adults engaging in physical activity. Our results demonstrate that when properly designed and supervised, square dance can be safely implemented even among older adults with relatively low baseline fitness levels. The group format provides social support and allows participants to observe and learn from others, while instructor supervision ensures proper technique and appropriate exercise intensity.

To contextualize our findings, it is useful to compare the effects of square dance with other evidence-based interventions for older adults. Meta-analyses examining various exercise modalities have found that multicomponent exercise (combining aerobic, strength, balance, and flexibility training), tai chi, and aerobic exercise all produce beneficial effects on physical and mental health in older adults (34). Our square dance intervention, which inherently incorporates aerobic activity, balance challenges, and flexibility training, produced effect sizes comparable to or exceeding those reported for these other modalities across multiple outcome domains. For depressive symptoms, a meta-analysis by Schuch et al. (35) reported an overall standardized mean difference of approximately−0.51 for exercise interventions in older adults with depression, while our study observed an effect size of d = 0.58 for the GDS-15. For functional mobility as assessed by the TUG test, network meta-analyses have reported standardized mean differences ranging from approximately−0.4 to−1.0 for various dance interventions compared to controls (27), consistent with our observed effect size of d = 0.71.

These comparisons suggest that square dance produces benefits of similar or greater magnitude compared to other established exercise interventions, while offering practical advantages in terms of accessibility, cost-effectiveness, cultural appropriateness, and participant enjoyment. The group-based social nature of square dance may provide additional psychosocial benefits beyond those achieved through individual exercise programs.

This randomized controlled trial demonstrated that a 12-week community-based square dance program significantly improved multiple health domains in older adults, including physical function, cardiovascular health, mental wellbeing, and quality of life, with high adherence, strong feasibility, and no serious adverse events. An important consideration in interpreting the findings is the predominantly female composition of the study sample. Previous research suggests that physiological and psychological responses to physical activity may differ between men and women, potentially influenced by hormonal, metabolic, and psychosocial factors. In particular, hormonal variations in older women may influence cardiovascular adaptation, mood regulation, and exercise responsiveness. Therefore, the findings may be more directly applicable to older female populations, and caution is warranted when generalizing to male participants. Strengths include its rigorous design, comprehensive multidimensional assessments, and consistent findings across analyses, though limitations such as short intervention duration, limited cultural generalizability, predominantly female participation, lack of cognitive assessments, and absence of objective intensity monitoring warrant caution.

The study highlights the need for long-term follow-up, cost-effectiveness analyses, mechanistic investigations, dose-response research, cultural adaptations, multimodal intervention combinations, and implementation studies to enhance scalability and sustainability. Practically, square dance emerges as a low-cost, safe, culturally rooted, and enjoyable intervention that healthcare providers, policymakers, and community organizations can promote to support healthy aging, while older adults may adopt it to improve physical and mental health. Overall, the findings position square dance as an effective, accessible, and scalable strategy for enhancing wellbeing in aging populations, underscoring its public health relevance and the importance of future research to optimize and expand its application. A potential limitation of this study is the unequal contact time between the intervention and control groups. Participants in the square dance group engaged in regular supervised sessions three times per week, whereas the control group received only limited health education sessions. This difference in exposure may have introduced attention and social interaction effects, which could partially contribute to the observed outcomes. Therefore, it is not possible to fully disentangle the effects of structured physical activity from those of increased social engagement. Future studies should consider using attention-matched control groups to better isolate the specific effects of the intervention.

## 5 Conclusions

This study suggests that participation in a 12-week community-based square dance program is associated with improvements in physical function, cardiovascular health, and psychological wellbeing among older adults. The high adherence, safety, and feasibility observed in this study support its potential as a practical community-based intervention. However, these findings should be interpreted with caution, and further large-scale randomized controlled trials with longer follow-up periods are needed to confirm the observed effects and establish causality.

## Data Availability

The original contributions presented in the study are included in the article/supplementary material, further inquiries can be directed to the corresponding author.
